# Multiple Realities and Hybrid Objects: A Creative Approach of Schizophrenic Delusion

**DOI:** 10.3389/fpsyg.2018.00107

**Published:** 2018-02-13

**Authors:** Michel Cermolacce, Katherine Despax, Raphaëlle Richieri, Jean Naudin

**Affiliations:** ^1^Department of Psychiatry, Hôpital Sainte-Marguerite, Aix-Marseilles University, Marseilles, France; ^2^Laboratory of Cognitive Neurosciences (LNC, Centre National de la Recherche Scientifique 7291), Aix-Marseilles University, Marseilles, France; ^3^Cross-Clinical Thinktank, Hôpital Sainte-Marguerite, Marseilles, France; ^4^Laboratory of Public Health (Health, Chronic Diseases and Quality of Life, EA 3279), Aix-Marseilles University, Marseilles, France

**Keywords:** schizophrenia, delusions, perspectivism, double book-keeping, phenomenology

## Abstract

Delusion is usually considered in DSM 5 as a false belief based on incorrect inference about external reality, but the issue of delusion raises crucial concerns, especially that of a possible (or absent) continuity between delusional and normal experiences, and the understanding of delusional experience. In the present study, we first aim to consider delusion from a perspectivist angle, according to the Multiple Reality Theory (MRT). In this model inherited from Alfred Schütz and recently addressed by Gallagher, we are not confronting one reality only, but several (such as the reality of everyday life, of imaginary life, of work, of delusion, etc.). In other terms, the MRT states that our own experience is not drawing its meaning from one reality identified as *the* outer reality but rather from a multiplicity of realities, each with their own logic and style. Two clinical cases illustrate how the Multiple Realities Theory (MRT) may help address the reality of delusion. Everyday reality and the reality of delusion may be articulated under a few conditions, such as compossibility [i.e., Double Book-Keeping (DBK), in Bleulerian terms] or flexibility. There are indeed possible bridges between them. Possible links with neuroscience or psychoanalysis are evoked. As the subject is confronting different realities, so do the objects among and toward which a subject is evolving. We call such objects Hybrid Objects (HO) due to their multiple belonging. They can operate as shifters, i.e., as some functional operators letting one switch from one reality to another. In the final section, we will emphasize how delusion flexibility, as a dynamic interaction between Multiple Realities, may offer psychotherapeutic possibilities within some reality shared with others, entailing relocation of the present subjects in regained access to some flexibility via Multiple Realities and perspectivism.

## Introduction

Delusions are currently held, as in the Diagnostic and Statistical Manual of Mental Disorders, (American Psychiatric Association, 2013), to be “*a false belief based on incorrect inference about external reality that is firmly held despite what almost everyone else believes and what constitutes incontrovertible and obvious proof of evidence to the contrary*” (p. 819). Indeed, a clinical specificity of delusion, regarding schizophrenia, is that it can have stabilized on permanent or recurring themes.

Yet another trait about delusion is that it is made up from some underlying activity, whether straightaway hallucinatory or derived from intuition. Jaspers called this: the “atmosphere” of delusion. Such fundamental delineation was emphasized on the occasion of the centenary of Jaspers' “*General Psychopathology*” (Jaspers, 1963). In a recent editorial, Maj insisted on the necessity to bridge the gap between the DSM and Jaspers' approaches (Maj, 2013).

This is a crucial epistemological challenge: that of a possible (or impossible) continuity of delusional atmosphere and ideas with normal experience. Delusional activity does not necessarily result from a radical modification of experience and may stand in continuity with normal experience. Yet the development of delusional ideas may result from some modification of potentially altered cognitive processes.

The issue of delusion may also be viewed from a perspectivist angle, as we shall see in the *Multiple Reality Theory* (MRT). This allows for cross-clinical approach (or cross-disciplinary approach to a clinical situation) to what may be called *Hybrid Objects* (HO), by extension from Latour (1991), that is: some objects from everyday life, which have a specific place within delusion, being part of several perspectives or realities at a time. Such objects can operate as *shifters*, i.e., as some functional operators letting one switch from one perspective (or world, universe, or reality) to another.

In this article, we mean to:

1. define the specific logic of the reality of everyday life, of imaginary life, the reality of delusion and of individual myth-making, therefore applying MRT to the reality of delusion, raising the question of Double Book-Keeping (DBK);
2. show how such realities are intricate, allowing for bridges from one to another within a third-party reality such as HO are likely to provide;
3. view how such bridges and HO may account for the flexibility of delusion by operating in some transitional intersubjective space-time;
4. study how these potentialities may be used as dynamic source for psychotherapeutic action.

From an epistemological point of view, we have a triple reference here:

First, a phenomenological one, as we rely on a clinical method aiming at suspension of any pre-established form of determinism. Moreover, other aspects of MRT are inherited from phenomenology. For instance, everyday reality may refer in phenomenological terms to the “*axioms of daily life*” (Straus, 1949), or to an attachment to a primordial reality, primarily related to an intersubjective world (Blankenburg, 2001).

Secondly, a psychoanalytical one, as this approach seems relevant to an exploration of the dynamic nature of intersubjective relationships and the resulting production of symptoms.

Thirdly, a constructivist/pragmatic reference may also be appropriate here. Since only theories and interpretations accounting for a deeper comprehension of experience, chain sequences of living experience and the jumps implied, can provisionally contribute to the fluidity and continuity of consciousness. In doing so, they tend to bridge the gaps of explanation and understanding and their own limits. Such third-party representations favor recovery, and are therefore welcome.

## Multiple reality theory (MRT)

The main theoretical reference is the MRT as developed by Schütz (1964) and addressed by Gallagher (2009): we are not confronting one reality only, but *several*. Therefore, the MRT participates in the making up of the sense of reality: starting from existing observation and theoretical milestones, we focus on the possible (or impossible) evolution of the connections between these realities, and what favors them.

Indeed, the MRT points out the notion of *perspectivism*, stating the necessity to define at least two different minimal perspectives, self and other. Grammatical persons (1-2-3, Singular/plural) and space-time language markers (here or there/today, yesterday, tomorrow) are essential to the construction of these categories, with a predominance of “here and now” (Naudin et al., 2000; Guigo-Banovic et al., 2003).

*Multiple Realities* proper refer to Alfred Schütz's views that we are confronting several types of realities, and not only one. In other terms, MRT states that our own experience is not drawing its meaning from one reality identified as *the* outer reality, that of the senses and perceived objects, but rather from a multiplicity of realities, each with their own logic and style. According to such MRT making Schütz one of the first constructivists, the reality of the senses, being also the reality of work and everyday reality, stands above all other types of realities as the primary or paramount reality from which all others proceed.

In this perspective the meaning of things is closely related to the specific logic of the reality in which they appear, and a thing may be grasped differently from several realities at the same time. So is everyday reality essentially rooted in perception, perceiving the world from the place where my body is, as a source of perception and movement, “*the null-point”* or “*point zero”* from which I can take part in the world, grasp things and meet people. Actually, paramount reality is currently known for short as “Reality” (Schütz, 1964) and will henceforward be mentioned as such. Time and space are inseparably bound together, implying some reciprocity of perspectives in a perspectivism rooted in the physical source and living embodiment we identify as self (Cermolacce et al., 2007; Fuchs and Schlimme, 2009).

So is there a dynamic dimension in MRT, as pointed out by Gallagher (Schütz, 1964), combining: (1) the attraction force of objects and related beliefs in the reality of delusion, (2) a weakness in the attachment to reality (i.e., the loss of vital contact, weakness of symbolic law in psychoanalysis, and weakening of minimal self in phenomenology).

The most recent and crucial contribution on MRT consists in a recent publication by Gallagher (2009), who retraces the development of the notion of MRT. A first origin of this notion was addressed by William James, who introduced the idea of possible “sub-universes.” Schütz further developed the concept of multiple realities, sub-universes or finite areas of meanings, against the hypothesis of “*one unified world of meaning that is objectively defined, in a view from nowhere*” (Gallagher, 2009). In order to exemplify how multiple realities can be articulated with paramount (or everyday) reality as defined by both James and Schütz, Gallagher suggests to consider several kinds of multiple realities that we frequently engage in, such as dream reality, fictional reality, or virtual reality. If I dream, I read a novel, I go to the cinema or if I play a videogame or immerse myself into a virtual world, I am somehow taken away from everyday reality. In such other realities, I can access a changed variation of myself, in an embodied and pragmatic way. As Gallagher emphasizes it, in the case of a vivid immersion, my engagement to a dream, a fictional or a virtual reality can be “body and soul.” In other terms, my presence to the world can be modified, and the transitions from one reality to another cannot be reduced to adopting an alternative set of beliefs or values. Such changes are first experienced in an existential way, “involving a transformation of background familiarity and of the sense of reality” for either entering or leaving various realities (Gallagher, 2009).

Gallagher suggests that entering a delusional reality may be described in the perspective of MRT. The reality of delusion itself allows for some specific logic: it does not go by the rules of perspectivism, and presents a potential for ubiquity, as persons with delusion may be both here and there at the same time, act and communicate from a distance, feel frequently observed while others are mind-reading their innermost secrets and giving them orders, anticipating their actions, knowing their intentions in advance. To them, the other may be neither seen nor grasped but given at once, even acting beforehand, even reversing the very movement of intentionality (Ogawa and Naudin, 1986; Naudin and Azorin, 1997; Fuchs and Schlimme, 2009). In hallucination, the other is not some actual *alter ego* but non-self, pure Otherness or alterity that the person with delusion will eventually never get to know anything about, other than being forcefully made to experience it.

In such perspective, discourse—involved in the narrative experience—may be defined as the Multiple Realities (MR) in which the cultural codes shared in everyday life and personal developmental acquisitions and typifications are organized.

The MRT, as reactualized by Gallagher, is not devoid of theoretical connections with psychopathological views developed by classical authors, such as Bleuler or Jaspers.

As in schizophrenia, delusional experience appears to exist alongside apparent rational thinking without conflict, Bleuler introduced the notion of DBK.

DBK emphasizes a mode of functioning on at least two different planes, as some non-delusional everyday reality may be observed to co-exist parallel to the reality of delusion, as DBK would in accountancy. Sass proposes to reconsider the notion of DBK initially introduced by Bleuler (Sass, 2014), and also suggested the terms “*Double registration”* or “*Double orientation”* to describe the coexistence of an absolute delusional certitude alongside a possibility for a shareable, practical everyday world orientation. In other words, patients suffering from schizophrenic delusion are somehow still able to cope with everyday reality, in spite of the *incomprehensibility* and *incorrigibility* of their delusional stance, as though delusion were untrue or irrelevant. The coexistence of both delusional and everyday realms implies to a certain degree the *inconsequentiality* of delusional experience. DBK may also partly explain why current research on insight, without taking such possible coexistence into account, may fail to give a consistent and specific model of schizophrenic delusion (Henriksen and Parnas, 2014). In our clinical examples, we shall explore the modalities allowing one to switch from one reality to another. In doing so, we address the question of whether they are each permanently accessible.

In his “General Psychopathology,” Jaspers considers reality as characterized by a set of intersubjective conventions that are coherent in terms of spatial and temporal structures. In normal experience, this coherence allows us to give meaning to reality, to access psychological comprehension (Jaspers, 1963). In schizophrenia, Jaspers postulates a breach in these comprehension links, which he called schizophrenic “process.”

According to Jaspers, this very notion of process is the reason why schizophrenic delusions (“delusions proper”) are fundamentally distinct from non-schizophrenic delusions such as in paranoia or in affective disorders (“delusion-like ideas”). Thus, schizophrenic delusions would steer clear of many attempts of psychological understanding. In a Jaspersian view, explanation of schizophrenic delusions would not imply equivalent models than for delusion-like ideas, from either neuroscientific or psychoanalytical approaches.

Reality and the reality of delusion, although exclusive one of the other, are not incompatible but *compossible;* they may therefore be articulated under conditions such as some third-party reality, transitional intersubjective space, or HO. If not, the different realities remain *in-compossible* in a situation of inflexibility of delusion, as in the clinical (counter-) example below.

## Clinical vignette KS (1): the rosary and knife or motives in delusion not eligible as HO

KS, 52, the elder of 2, is suffering from paranoid schizophrenia. The first delusional episode took place on starting reading Law at 20. KS is well-read, a fond reader of accursed poets and the bible, keen on politics and history. He is single, close to his parents, French natives forced to leave Algeria on decolonization before he was born.

He presents a delusion of control, with strong and frequent visual and auditory verbal hallucinations along two main themes: the Cold War and his identification with Christ. These themes are intricate in a reality of delusion wherein he may have successive identities and enemies, mainly being Christ working miracles, converting people, fighting communists and Muslims alike, as a crusader would; he carries a crucifix, rosary and knife, just in case the enemy refused to be converted. He deeply suffers too, harassed by voices persecuting him via comments and orders. He can communicate with God directly.

Although suffering from delusion and frequently hospitalized, he lives with his family in a shared reality, having meals, baby-sitting nephews, taking care of sick relatives, being concerned with his father's health and going on holiday with them. He does not work and is granted a pension in compensation for his handicap. During hospitalizations, he is familiar with life in the care unit and appeased by the rhythm set by medical staff. He will easily jump to conclusions since the inflexibility of his own belief and delusional certitude may serve as immediate response to his anxiety (Garety et al., 2005).

Informal support from caregivers is a determining factor favoring the flexibility of delusional beliefs (Jolley et al., 2014). Therefore medical staff discuss with him his current attempt at conversion of a secretary by entering her office holding rosary and knife, to her immediate fright. Nurses have since then been asking him how, he thought, others might be viewing him and the situation.

We can see here that although the crucifix, rosary and knife are borrowed emblems from the reality of the crusades, they cannot function as HO and do not belong to any shareable Reality (of discourse): they have been used to break into the secretary's current reality of work where they do not belong, and did not allow for any third-party intersubjective space or reality.

Indeed, as we have seen *a contrario* from KS (1) clinical situation, all objects are not eligible as HO. They cannot stand as such if they belong to the reality of delusion only and not to two at least, including some shared reality at the same time. Whereas delusion is in itself viewed as clearly *solipsistic*, if the second condition be met concerning shared objects, it may be interesting from a clinician's perspective to find out about these HO, in order to resume or facilitate discussion with the person with delusion, or it may even be the occasion for therapeutic intervention.

Hence the clinical relevance of MRT toward some dynamic comprehension of delusion, showing us how some persons presenting an episode of delusion are still confronting several realities, some of which seem to be incompatible, while others seem to allow for a function as *transitional* realities. A dynamic approach to the links between the reality of delusion and Reality gives better insight into the flexibility of delusion and the part of intersubjectivity that some imaginary, linguistic or fantasy third-party reality may play in it.

According to Gallagher, the Multiple Realities paradigm provides an alternative to main models of delusion inherited from neurosciences, mainly from a cognitivist perspective (Gallagher, 2009; Sass and Byrom, 2015). Empirical studies on delusion aim to explain delusional processes by exploring several and distinct kinds of delusional disorders, from neurological to psychiatric conditions. Current studies on delusions refer to two possible main accounts: the *rationalist* model (based on impaired rationality), or the *empiricist* model (based on unusual experience).

In the rationalist approach, delusion mainly deals with the alteration of reasoning, closely linked to a non-understanding of delusional content, and does not rely on abnormal perceptual experience (Campbell, 2001). Such approach puts the emphasis on top-down disturbances, and relies on some altered fundamental beliefs about the world. In its most radical version, perception is perfectly preserved, as initially suggested by Jaspers: delusion proper is “*direct experiences of meaning while perception itself remains normal and unchanged”* (Jaspers, 1963; Jones, 1999). In its softer and more frequently accepted version, the rationalist model envisages the possibility of perceptual alterations by top-down constraints, involved in the maintaining of delusion (Campbell, 2001).

Conversely, empiricist models of delusion imply a broadly preserved rationality, from totally intact (in its radical version) to secondary involved (in its moderate version). These models rely on a highly unusual experience, e.g., a perceptual experience. Delusional content is secondarily built or translated with a mainly rational development, and with an intact understanding of the meaning of its terms (Langdon and Coltheart, 2000; Bayne and Pacherie, 2004). Alternatively, progressive and intermediary models combine bottom-up and top-down constraints, by involving two distinct factors (first-order experience and high-order cognition) (Campbell, 2001; Gallagher, 2009).

The possibility for a subject to take up the effect of this dysfunction or the scientific explanations for it, into the subject's own perception of the world so that such personal experience may make sense, have led these therapies to aim at some narrative perspective. The advantage with MRT is that instead of focusing on a person's experience, it is focusing on the very structure of the confronted reality.

## Hybrid objects (HO)

In this line, we may understand how some elements making up the sense of Reality may be shared by several persons at the same time. As the subject is confronting different realities, so do the objects among and toward which a subject is evolving, belong to several realities at the same time. Within each of these realities, the object is given in some specific space-time signaled within discourse by the interplay of linguistic markers. The possibility for the subject to jump from one space-time to another or combine them together results from the connections established with an object that is making them compatible. We call such objects *Hybrid Objects* due to their multiple belonging.

The dynamic specificity of HO may account for the flexibility of delusion. These objects may be empirically located in the space-time of primary or paramount reality. But they may also be related to the specific space-time of the reality of delusion where perspectivism does not apply, where temporality, free from clock-time, may be indefinite, cyclical or reversible, where space, free from embodiment, may be spanned disregarding distances, measures or limits assigning everyone in the paramount reality of nature and regarding others, to some precise location, inside or outside.

Last but not least, HO are themselves included in a *third-party reality* placing them, as it were, at the center of a plot. This gives delusion in-the-making its own specific style and narrative structure. Such reality is usually rooted in the cultural background and its mood in the words or language particularities used to describe it. It suits the subject, being made from signs and providing a key for interpretation, or a different conducting thread from the one interconnecting the meaning of all things in Reality.

HO belong to several realities at the same time. They are bridging paramount and delusional realities, or paramount reality and the diverse realities which, according to Schütz's theory, proceed from it to some extent. Such HO are often making up a third-party reality, as fetiches can do in a religious reality, or technical objects in the scientific reality. They bear and crystallize a form of power over things, transcending the space-time dimensions of reality.

These objects are a possible space-time mediation of intersubjective relationship or relationship to the world. They may therefore prove helpful to the psychotherapist in relating to the person. In a psychoanalytical approach, they are related to Winnicott's concept of “experiencing” or “transitional” area.

Indeed, using space-time references, in terms of the logic of infant psychic development rather than mere chronology, D.W.Winnicott refers to a “transition *phase*” as an “intermediary *place*” between primary creativity and objective perception (based on the experience of Reality), also described as “the resting place of illusion.” This transitional area (which he specifies is neither inner nor outer space, from the infant's perspective), is the place for “*experiencing*,” the safe area “good-enough” parenting environment allows for, and the “basis of the initiation of experience.”

“Experiencing” contributes an “imaginative elaboration of function” in which “*play”* (as a dynamic factor for creative experience, interaction, and for making and testing hypotheses), and “*transitional objects*,” have a core function. Transitional objects are defined as these “*first not-me possessions*” elected (and named) to bear this “imaginative elaboration of function,” and are consequently eligible to represent for instance both actual exterior mother's breast and feeding, or an internalized one, before any sense of self vs. other. We feel this fluctuating and multiple belonging to be akin to the flexibility of later perspectivism, or to the creative potentialities of HO allowing for interactions in and out of MR, or between self and other in a shareable Reality (Winnicott, 1953, 1958).

These HO also allow for emotions to crystallize on something outside the subject's person and body. Therefore they require the person's power to be activated, and can be targeted with the intentionality of emotion, will or desire. Without any direct reference to Freud, Husserl calls it “pulsional reality.” The interplay of affordances meets the possible polarization of the subject's power over a given object within the relevant reality. Here, the term of affordances refers to Gibson's definition, as possible transactions between individuals and their environment (Greeno, 1994).

These objects are therefore also possible vehicles for emotions, turning them into affects by granting them rhythm, incarnation and temporality, according to the scale of each reality. This is a precondition for affective transference in psychoanalysis. Such is, in other terms, what takes place in the transitional area operating, via HO, a transferability of emotions and affects toward an object or from a person to another.

The existence of HO in the reality of delusion reveals a human possibility to be part of several realities at the same time, and for a subject shut up inside one reality only, to be set partly free via their interplay. So can we both account for the flexibility of delusion and for the possible conditions for a verbal treatment of it.

In the clinical cases described in this paper, we can see, beyond the limits of explanation, how with HO comprehension is not unconditionally necessary for a subject to be functional: the issue of meaning is no longer some insurmountable obstacle, provided such HO may open access to the question of functionality, i.e., to the different logical places involved and the possible operators allowing circulation between them. It is therefore quite useful to find out what functions as HO, and learn how to use them, i.e., let them teach us how they can be used for therapeutic aims.

## Clinical vignette KS (2): the telephone as *hybrid object*

We have chosen examples concerning *the telephone* as typical HO owing to its demonstration value. The issue at stake is to favor a patient's ability to move from one reality to another, promoting the emergence of creative solutions via idiosyncratic or poetic objects (as with surrealist poetic creation or poetic games).

We are calling such objects “*hybrid*” since, while a subject is self-asserting as an actor by intentionally targeting them, they are taking part in at least two realities at the same time, the reality of delusion and Reality.

The object here is a beige telephone at KS's disposal as in any hospital bedroom. The beige telephone from Reality turns into the “*red*” hotline (“*téléphone rouge”* for hotline in French), through which Stalin is communicating in the reality of delusion. So the telephone is in turn a linguistic object granting KS power in the reality of delusion, as the “red” hotline, or a simple tool, the ordinary beige object producing a simple tone when KS lifts up the receiver in front of others, a meaningless tone to all, that he interprets as some coded message. As his psychiatrist entered his room once with other staff while KS was hallucinating, claiming to be on the phone with Stalin, the psychiatrist replied: “*Please tell Mr. Stalin that you are busy and he must call back later.”*

This example shows how more flexibility can be obtained concerning delusional beliefs, playing on the *in-between* revealed and created by a HO. Of course, humor cannot be mentioned as some magic trick from a therapeutic toolbox (Gibbs, 2007); this is rather an illustration of a therapeutic mode of action based on spontaneous intervention, by no means to be viewed as some strategy. The operating mode consists of shifting from one reality to another *via* some third-party reality, in this case, from the reality of delusion into Reality *via* the imaginary reality of metacognitive representation (“*Mr Stalin”)*. The effect on the flexibility of delusional beliefs may be noticed as the belief is getting gradually modified. Indeed KS first answers that he is interpreting the coded message in the telephone tone, then puts an end to it saying: “*he must have hung down the receiver.”*

In a session with his psychotherapist, KS grants that Stalin would not do so if he were not so concerned with his father's health, being Stalin's way to weaken him at a time when he needed to be strong and react; at the end of the session, he proves to be preoccupied with his father's current condition without getting invaded with delusional ideas, thus demonstrating how some metacognition, i.e., thinking about delusional beliefs, may for a short while favor some recovery (Garety et al., 2005).

KS's telephone enables him to have a long-distance communication with Stalin who personifies the supreme soviet power. On one hand, as a “téléphone rouge (“red” hotline), it gives access to a third-party reality through which KS can do everything. On the other hand, turning beige and a banal technical object in Reality, it enables KS to escape from the current concrete patient/nurses relationship. The telephone therefore materializes KS's possible power over things, according to the typical affordances of each reality it belongs to.

As every French person knows from school years and the media, dating back to cold war years between Soviet Union and the West, presidents are supposed to have direct phone contacts likely to alleviate difficulties via what is viewed as a “hotline,” similarly to the ones provided for users and consumers to solve their technical or administrative problems. Just as “red tape” is the English for “bureaucracy,” “the red telephone” (“le téléphone rouge”) is French for hotline.

Clinical examples of the use of the telephone among patients with verbal hallucinations show us how patients lift up their mobile phone to the ear while hearing voices. The evident benefit of such creations (“*trouvaille”*) is to minimize the social impact of verbal hallucination and, while it belongs in the reality of delusion, give it scope to be expressed in the pseudo-normality and shared cultural behavior of appearances, in a (seemingly) shared Reality. The telephone therefore seems to be liable to make up a possible interface between Reality (as a banal object, with implications concerning make-believe, and the social link) and the reality of delusion (as a modern technological object implying different degrees of familiarity and expertise or strangeness).

In line with this notion of possible interface, we can see in the film “*Matrix”* how the telephone, or a mere telephone ring, function as “*shifter*” letting the protagonists from one reality into another circulating between (supposedly) real and imaginary worlds. This “*motif* ” signals, in Carrollian or Joycian manner, the shifting from a world and space-time into another.

## The flexibility of delusion

The flexibility of delusion allows the creation or re-creation of some *intersubjective space*, which may be envisaged as some dynamic interaction of MR. This flexibility implies:

- enclaves from one reality into another;
- possible switching from one reality to another;
- mediation of some third-party reality.

This raises several questions of some possible therapeutic action with delusion flexibility. May HO which belong to two or more realities change in status (e.g., literal or figurative value)? Given what circumstances or conditions? Is there any possibility to circulate between one world or reality and another? If we hold delusion flexibility as some dynamic interaction between MR, then, disregarding content and theme, is it possible to accompany or induce some adaptive flexibility?

In the journey of Don Quixote (Schütz, 1964), while the hero is claiming a shaving dish to be a helmet in his reality of chivalry, his squire Sancho Panza finds some compromising solution by calling the object a “*helmet-shaving-dish,”* i.e., a pure language object establishing “*a sub-universe of discourse*” (p. 142) and allowing a dangerously conflictual situation to be disentangled. This type of object, both material and linguistic, is indeed to be frequently found as an active part of the dynamics of hallucination, and a pivot from which it is materially anchored in Reality.

So does KS link an object, a *telephone*, for everyone to see in Reality, with a possibility to communicate with invisible almighty speaking beings that are only present in the solipsistic reality of delusion.

This HO, owing to its quadruple belonging (Reality, imaginary reality, reality of delusion and the reality of the history of the Cold War), is also *a linguistic object*. The “*red hotline”* enables KS to get out of the tricky situation of jointly having to resume control of his experience, live a daily life among other people, and bridge the gap between visible and invisible, material and imaginary. And also go on believing one can proudly live in a world that is now over, in which his position may be pronounced invincible and death not opposed to life, as the living and the dead are coexisting in the present of hallucination.

Such an object functions as a bridge between a reality which has no justification beyond itself and cannot be corrected by experience, and the essentially perspectivist Reality. It functions as a personal creation to control and absorb the emotions raised by the strangeness of hallucination and alleviate its social impact. We can see here the same dynamics as in the Bleulerian notion of DBK.

## Clinical vignette: ZG and the second-hand bookseller's stand

The following clinical case enables us to pinpoint the type of reality confronting the subject and how the subject is placed on a double level facing delusional solipsistic reality and Reality. We can then grow aware of different stages in the subject's discourse, which can be defined with reference to the type of perspective, or reality where the subject stands.

ZG, 43 years, an only son, is a second-hand books and records seller with a registered outdoors stand, granted municipal license as one of a few privileged authorized professionals are. His parents and family reality is organized with reference to the themes of filiation and heritage; they have a nearby bookstand as ZG shares the same professional reality as his parents and, by extension, booksellers owning neighboring bookstands, passers-by and all potential customers. Defining his own place and everyone's function in these two realities is, to him, in every reorganizing stance of his existence, some permanent concern. He himself describes his professional activity as structured by two complementary functions: *selling* and *buying*, where he knows he has some facility at “manipulating” others, which is the source of a feeling of guilt. The second-hand bookstand, boxes and records making up the family, professional or administrative realities, are the setting where ZG's fight for acknowledgment may find some inscription in several realities at the same time.

He was diagnosed with schizo-affective psychosis during his first hospitalization owing to a 6 month's delusional episode made up of barely comprehensible ideas of filiation, grandeur and persecution. When he is not feeling well, which is often triggered by the arrival of some nearby rival, soon turning into a persecutor, he is disoriented; it seems then that only delusion can provide a set of clues to a world or reality of signs.

His delusional reality is two-fold: (i) a *technical reality* in which he stands as an actor of business exchanges within the reality of trade, and (ii) a *mythical reality* in which he is rich, all-powerful and where plots are made up and he stands a victim.

According to family history, a year before her marriage and a few years before he was born, his mother used to be a prostitute whom his father has “*bought back,”* in a local historical context very much like the *Three-Penny's Opera* in which prostitution was territorialized and prostitutes supposedly depending on their “protector,” himself economically depending on them as his source of revenue and business. ZG refers to his mother's pre-marriage situation as penniless, insecure and precarious, also possibly raped at 16 by her supposedly violent father.

This raises aggressive insults, and questions ZG within some moral or religious reality in which his mother is now “straight” since she was de-blacklisted,” and in some administrative reality in which bookstands and prostitutes alike have to be registered. Having an honest work as established booksellers allows ZG and his mother a “new start in life,” moralizing and normalizing relationships with others.

We can list a number of realities in which he acts, alone or among others:

- the professional *reality of work* which Schütz views as *paramount*, here the trading reality where buying and selling are the basic strategies supporting him, among the three pillars of his paramount reality, alongside with family and administration;
- an *imaginary reality*, which ZG will acknowledge when he is not in an episode of delusion;
- the *reality of delusion* made up of signs and clues from the themes of filiation and money, in which he is threatened and his property is envied, and where he switches from vulnerability (and a need for protection) to powerfulness;
- the *administrative reality* in which registrations are delivered allowing for business practices (as bookseller or prostitute).

Two types of objects seem to achieve some specific transitional function allowing or facilitating the passage from one reality to another. Enclaved one in the other and mixed as the realities are where they appear, they allow for some circulation of affects from one to another:

- the bookseller's stand and boxes containing merchandise on display, calling for envy in the theater of professional exchanges;
- money, whose symbolic value is held to be essential, circulates from hand to hand, setting the value of merchandise and a regulation of exchanges, made desirable and warranting stability and consideration (Bauersfeld, 1968), like the records that ZG bought from a seller, that are causing sensation and are purchased by others as access to the mother's body once was. This object may be appreciated as rarity, and price creates consideration and pride, both in seller and buyer alike.

Indeed, we have another type of HO with the book-selling stand: it is for ZG a place for circulation, exhibition and exchange where his “records” are gathered and presented in boxes to be searched by passers-by and exchanged for money. There, as in an enclave in the midst of everyday life, the different realities and the reality of delusion are intricate while the bookseller's stand must have official administrative status via a contract warranting its usual place. And the “expenses” entailed both belong to Reality and to the reality of delusion.

The main point is that the HO, as the telephone did for KS (2), is maintaining a function of crystallization and space-time orientation, of substitute bridging a gap or compensating for a void where the subject might otherwise vanish. Such an “*as-if”* function, for want of stronger symbolical binding, is holding together some elements which would otherwise be disseminated, lost to some disaffected world with no possible orientation or appropriation from a subject remaining unable to grasp it. When difficulty arises, then a lack of solid binding between things and between words and things is revealed, leaving room for some open void on which delusion is building up as renouncement or loss of natural contact with Reality. Such an issue has been quite thoroughly discussed by Sass in an article describing how reflexivity and delusion are complementarily compensating for a failing in *ipseity*, which is the failing in minimal self at the core of the schizophrenic experience (Sass and Byrom, 2015).

It is to be noted that exchanges are clearly regulated for ZG by money and law, referring to his past “*shameful”* experience, on learning that his mother was a prostitute before he was born and accusing his father, who had “*bought her back,”* of being responsible for passing no “*heritage*” or “*solid values”* over to him.

In ZG's case, professional and administrative realities, via the bookseller's stand, are equivalent for some transitional space between the reality of delusion and Reality, where the substitutive function is fully working (as when the sales make up for expenses) and delusion can recede to the back of the stage.

We may call “*plasticity”* such minimal potential for evolution, in a broader sense than neuroplasticity. Plasticity allows for flexibility disregarding causes or conditions, which are dealt with by cognitivist and scientific approaches as a field of their own.

The persistence of delusion is in itself a clinical issue that we can view on its own, disregarding a joint review of its plasticity. Concerning the development of delusion, persistence and plasticity stand as two dynamic vectors. We clearly have to grasp this question from either end so that it does not remain a vague or purely theoretical issue. So have we meant to grasp it here, in the HO to be commonly found both in delusion and in everyday life.

We suggest MRT can be compatible with neuroscientific and empirical perspectives, concerning several issues:

- First, as synthetized by Gallagher (2009), bottom-up (empirical) or top-down (rationalist) approaches to delusion are particularly apt to MRT modelization, though we insist on differentiating the diagnostic specificity of the distinct types of delusion.
- Secondly, several studies on schizophrenia have recently addressed the links between disturbed salience and delusions associated with self disorders (Nelson et al., 2014), and this hypothesis stands in favor of further exploration of possible links between salience and MRT perspectives.
- Lastly, patients with schizophrenia may present difficulties in cognitive flexibility, in terms of mental rotation. Flexibility, bridging ego-centered perspective (about self and the surrounding world) and allo-centered perspective (non-related to first person perspective), would be impaired and associated with delusional experience (Frith and de Vignemont, 2005).

Here, MRT may be explored by using discourse analysis tools, collecting narratives about self (Parnas et al., 2005) and world experience (Sass et al., 2017), or focusing on discourse markers (Maj, 2013).

## Conclusion

We have meant to illustrate in these clinical references how the Multiple Realities Theory (MRT) may be used to approach the reality of delusion, by investigating the issue of DBK and the flexibility of delusion. There are indeed some shifters or bridges between them, via HO, being either technological or practical or language objects.

The minimal condition for this dynamic function of HO is to have two (at least) differentiated levels, perspectives or realities making up the MR, while the HO belonging to these two (at least) realities may become functional and shareable in some third-party reality hosting them, however temporarily.

As a conclusion, the notions of MRT, DBK and HO, in the context of psychotherapeutic treatment of schizophrenic delusion, emphasize the core function of intersubjectivity to experience a possibility for evolution and creative potentialities for psychic mobility and interplay, *via* such objects. HO allow for reorganization within some reality shared with others, entailing relocation of the present subjects in regained perspectivism.

## Author contributions

MC works as clinician in Marseilles' University Department of Psychiatry, France. His main research interest concerns early stages of schizophrenia, at both subjective (the phenomenological approach to self-disorders) and objective levels (electrophysiology). KD, graduate of the École Normale Supérieure, is psychoanalyst. She also conducts hospital supervisions of work situations and professional practices. Her main research activity involves building cross-clinical therapeutic and theoretical approaches; she leads a regular seminar on a clinical approach to postmodernity. RR works as psychiatrist in Marseilles' University Department of Psychiatry, France. Her main research interest concerns treatment-resistant mental disorders (schizophrenia and depression) and neuro-stimulation. She is also affiliated to Schizophrenia Expert Center, Fondation FondaMental, France. JN is Professor of Psychiatry and co-leads Marseilles' University Department of Psychiatry, France. He is specialized in Phenomenological Psychiatry and Existential Analysis.

### Conflict of interest statement

The authors declare that the research was conducted in the absence of any commercial or financial relationships that could be construed as a potential conflict of interest.
